# Estradiol Preferentially Induces Progestin Receptor-A (PR-A) Over PR-B in Cells Expressing Nuclear Receptor Coactivators in the Female Mouse Hypothalamus^1^,^2^,^3^

**DOI:** 10.1523/ENEURO.0012-15.2015

**Published:** 2015-08-13

**Authors:** Kalpana D. Acharya, Sarah D. Finkelstein, Elizabeth P. Bless, Sabin A. Nettles, Biserka Mulac-Jericevic, Orla M. Conneely, Shaila K. Mani, Marc J. Tetel

**Affiliations:** 1 Neuroscience Program, Wellesley College, Wellesley, Massachusetts 02481; 2 Department of Molecular and Cellular Biology, Baylor College of Medicine, Houston, Texas 77030

**Keywords:** estrogens, female reproduction, progesterone, SRC-1, steroid receptor coactivator, ventromedial hypothalamus

## Abstract

Estrogens act in brain to profoundly influence neurogenesis, sexual differentiation, neuroprotection, cognition, energy homeostasis, and female reproductive behavior and physiology through a variety of mechanisms, including the induction of progestin receptors (PRs). PRs are expressed as two isoforms, PR-A and PR-B, that have distinct functions in physiology and behavior. Because these PR isoforms cannot be distinguished using cellular resolution techniques, the present study used isoform-specific null mutant mice that lack PR-A or PR-B for the first time to investigate whether 17β-estradiol benzoate (EB) regulates the differential expression of the PR isoforms in the ventromedial nucleus of the hypothalamus (VMN), arcuate nucleus, and medial preoptic area, brain regions that are rich in EB-induced PRs. Interestingly, EB induced more PR-A than PR-B in all three brain regions, suggesting that PR-A is the predominant isoform in these regions. Given that steroid receptor coactivator (SRC)-1 and SRC-2 are important in estrogen receptor (ER)-dependent transcription in brain, including PR induction, we tested whether the expression of these coactivators was correlated with PR isoform expression. The majority of EB-induced PR cells expressed both SRC-1 and SRC-2 in the three brain regions of all genotypes. Interestingly, the intensity of PR-A immunoreactivity correlated with SRC-2 expression in the VMN, providing a potential mechanism for selective ER-mediated transactivation of PR-A over PR-B in a brain region-specific manner. In summary, these novel findings indicate that estrogens differentially regulate PR-A and PR-B expression in the female hypothalamus, and provide a mechanism by which steroid action in brain can selectively modulate behavior and physiology.

## Significance Statement

Progesterone acts in brain to influence neuroprotection, sexual differentiation, cognition, energy homeostasis, and reproduction. Many of these effects are mediated through the estradiol induction of two progestin receptor (PR) isoforms, PR-A and PR-B, which have distinct functions. However, due to the inability to distinguish these two PR isoforms at the cellular level, it is not known whether estradiol differentially induces these isoforms in brain. Using PR isoform-specific knock-out mice, we show that estradiol differentially regulates these PR isoforms in a brain region-specific manner. Furthermore, our data suggest that steroid receptor coactivator-2 preferentially functions in the estradiol induction of hypothalamic PR-A, but not in that of PR-B. These findings provide a mechanism by which progestin action in brain can selectively regulate behavior and physiology.

## Introduction

The sex steroid hormones estradiol and progesterone act in the brain to influence neurogenesis, neuroprotection, sexual differentiation, learning and memory, energy homeostasis, and reproductive behavior and physiology (Simerly, 2005; Brinton et al., 2008; Barha and Galea, 2010; Pfaff et al., 2011; Mani and Oyola, 2012; McCarthy and Nugent, 2013; Bless et al., 2014; Arevalo et al., 2015; Frick, 2015; López and Tena-Sempere, 2015; Willing and Wagner, 2015). Estrogens and progestins elicit many of these functions by binding to estrogen receptors (ERs) and progestin receptors (PRs), respectively (Conneely et al., 1986; Conneely et al., 1987; Tsai and O'Malley, 1994; Mangelsdorf et al., 1995; Tetel et al., 2009). ERs and PRs have traditionally been thought of as ligand-dependent transcription factors, which regulate physiology and behavior primarily through classic genomic mechanisms (Blaustein, 2008; Tetel et al., 2009; Tetel and Pfaff, 2010; Mani and Oyola, 2012). In the classic genomic mechanism of action, the steroid binds to its receptor and the activated receptors bind to the promoter region of target genes to mediate gene transcription (Kraus et al., 1994; Stanisić et al., 2010). However, these receptors can also elicit profound effects on behavior and physiology by functioning independent of the ligand on DNA or at the membrane to rapidly activate cytoplasmic signaling pathways (Kelly and Rønnekleiv, 2008; Vasudevan and Pfaff, 2008; Mani et al., 2012; Sinchak and Wagner, 2012).

A classic example of an estrogen-responsive target gene is the progestin receptor gene (*Pgr*), which codes for PR protein (Tetel and Lange, 2009; Mani and Oyola, 2012). In humans and rodents, there are two major PR isoforms, the N-terminally truncated PR-A and full-length PR-B, which are expressed in brain, and are transcribed from the same gene but are regulated by different promoters (Conneely et al., 1987; Kastner et al., 1990; Savouret et al., 1991; Kraus et al., 1993; Hagihara et al., 1994; Kraus et al., 1994). Except for the PR-B upstream sequences (BUS region of ∼165 aa), both PRs are identical and contain a variable N-terminal regulatory domain, a highly conserved DNA binding domain, a C-terminal ligand-binding domain (Dobson et al., 1989; Meyer et al., 1990), and two activation function (AF) domains that interact with other transcription factors (Sartorius et al., 1994; Tetel et al., 1999). The PR-B-specific AF3 domain is responsible for the stronger transcriptional activity of human PR-B than of PR-A (Sartorius et al., 1994; Giangrande et al., 1997; Hovland et al., 1998; Tung et al., 2006).

Estradiol-induced PRs are expressed in several regions of the rodent brain, including the medial preoptic area (MPA), ventromedial nucleus of the hypothalamus (VMN), and arcuate nucleus (ARC; MacLusky and McEwen, 1980; Blaustein and Turcotte, 1989; Pleim et al., 1989; Lauber et al., 1991; Moffatt et al., 1998; Kudwa and Rissman, 2003), which are important brain regions involved in energy homeostasis and female reproductive physiology and behavior (Davis et al., 1982; Guerra-Araiza et al., 2003; Mulac-Jericevic and Conneely, 2004; Brinton et al., 2008; Conneely, 2010; Mani and Blaustein, 2012; Bless et al., 2014; López and Tena-Sempere, 2015). Peripheral tissues, including the ovary, uterus, and mammary gland, also express both PR isoforms (Clarke et al., 1987; Shyamala et al., 1990; Conneely et al., 2001, 2003; Mote et al., 2001). PR-A and PR-B exert distinct roles in a tissue- and species-dependent manner. In brain, while both PR-A and PR-B are necessary for the full expression of progestin-facilitated lordosis in female mice, PR-A appears to contribute more to this behavior (Mani et al., 2006). In certain human cell lines, PR-B is a stronger transcriptional activator than PR-A, and PR-A represses the transcriptional activity of PR-B (Vegeto et al., 1993; Wen et al., 1994; Giangrande et al., 1997). In canines, PR-B greatly differs from human PR-B in the N-terminus and mediates a weaker transcriptional activity compared with the human PR-B (Gracanin et al., 2012, 2014).

Steroid receptors regulate the transcription of target genes through interaction with nuclear receptor coregulators that consist of nuclear receptor coactivators and corepressors (Rosenfeld et al., 2006; Lonard and O'Malley, 2012). Nuclear receptor coactivators can dramatically enhance receptor function through histone acetyltransferase activity, stabilization of nuclear receptors, and alternate RNA splicing (Li et al., 2003; Dowhan et al., 2005; Rosenfeld et al., 2006; Johnson and O'Malley, 2012). One important group of coactivators in nuclear receptor action is the p160 family of coactivators, which includes steroid receptor coactivator (SRC)-1, SRC-2, and SRC-3 (Lonard and O'Malley, 2012). SRC-1 and SRC-2 are important in energy homeostasis, circadian rhythm, sexual differentiation of the brain, stress, and reproductive behavior (Auger et al., 2000; Charlier et al., 2006; Molenda-Figueira et al., 2006; Winnay et al., 2006; Lachize et al., 2009; Zhu et al., 2013; Stashi et al., 2014a,b). For example, these coactivators mediate ER and PR action in brain (Tetel et al., 2009; Tetel and Acharya, 2013), and are highly expressed in the VMN, ARC, MPA, hippocampus, and amygdala (Meijer et al., 2000; Tetel et al., 2007; Bian et al., 2011; Tognoni et al., 2011). The majority of 17β-estradiol benzoate (EB)-induced PR cells in the VMN, ARC, and MPA of female mice express both SRC-1 and SRC-2 (Tognoni et al., 2011), suggesting that these cells are functional sites of interaction between steroid receptors and coactivators in brain. In further support of this interaction, SRC-1 and SRC-2 from rodent brain physically associate with both ER subtypes (ERα and ERβ) and PR isoforms in a ligand-dependent and brain region-specific manner (Molenda-Figueira et al., 2008; Yore et al., 2010). Finally, decreasing SRC-1 or SRC-2 protein expression in the hypothalamus reduces EB-induced PR expression in the VMN (Apostolakis et al., 2002; Molenda et al., 2002), and ER- and PR-dependent aspects of female reproductive behavior (Molenda et al., 2002; Molenda-Figueira et al., 2006).

PR-A and PR-B have distinct and important functions in reproductive behavior and physiology (Conneely et al., 2001, 2003; Mani et al., 2006; Guerra-Araiza et al., 2009). However, because the truncated PR-A is transcribed from the same gene as PR-B, and is thus identical in amino acid sequences, the use of antibodies or *in situ* hybridization to investigate the expression of these PR isoforms at the cellular level in brain has been severely limited. To overcome this limitation, the present study, for the first time, used PR isoform-specific null mutant mice (PRAKO and PRBKO) to test the hypothesis that EB-induced PR-A and PR-B are differentially expressed in the female hypothalamus. Furthermore, in order to explore the potential role of SRC-1 and SRC-2 in the EB-mediated expression of the PR isoforms, the coexpression of these coactivators with PR-A and PR-B in brain was investigated.

## Materials and Methods

### Animals

Female heterozygous (PRA^+/−^ and PRB^+/−^) and male homozygous (PRAKO and PRBKO) mice were obtained from the Conneely laboratory at Baylor College of Medicine (Houston, TX; Conneely et al., 2001; Mulac-Jericevic et al., 2003). Subsequent generations were bred and housed in the Wellesley College Animal Facility (Wellesley, MA). All animals were housed in groups of three to six mice under a 12 h light/dark cycle. Food and water were available *ad libitum*. All animal procedures were approved by the Institutional Animal Care and Use Committee of Wellesley College and were conducted in accordance with the National Institutes of Health *Guide for the Care and Use of Laboratory Animals*.

### Ovariectomy and hormone treatments

PRAKO and PRBKO null mutant females and their respective wild-type (wt) female littermates (*n* = 6-10/per group) were used for the study. Mice were bilaterally ovariectomized between 8 and 10 weeks of age under 1.5% isoflurane. One week following surgery, mice were injected subcutaneously with 1 μg of EB (dissolved in 100 μl of sesame oil) to induce PR or vehicle (100 μl of sesame oil).

### Perfusion and tissue collection

Forty-eight hours after EB or vehicle injections, mice were killed by an intraperitoneal injection of Fatal Plus (sodium pentobarbital; 390 mg/ml, 100 μl). Mice were perfused with 8 ml of 0.9% saline for 1 min, followed by 4% paraformaldehyde (w/v) in 0.1 m phosphate buffer (PB), pH 7.2, for 8 min at a flow rate of 8 ml/min. Brains were collected and post-fixed in a 4% paraformaldehyde solution at 4°C for 3 h. The fixed brains were transferred and stored in a 20% sucrose solution in 0.1 m PB, pH 7.2, for 48 h and cut into 40-μm-thick coronal sections from the MPA through the hypothalamus using a freezing microtome. The sections were stored at −20°C in cryoprotectant until they were used for immunohistochemistry.

### Triple-label immunohistochemistry

Triple-label immunohistochemistry was used to identify EB-induced PR-A-immunoreactive (PRA-ir) and PRB-ir cells that coexpress SRC-1 and/or SRC-2 in the VMN, ARC, and MPA. Brain sections were washed with 0.1 m glycine in 0.05 m Tris-buffered saline (TBS) for 30 min followed by a 20 min wash in 0.5% sodium borohydride (w/v) in TBS to remove excess fixative from the tissue. Following additional washes in TBS, the sections were incubated in a TBS solution containing donkey-anti-mouse IgG (Jackson ImmunoResearch) for 90 min to block nonspecific binding sites for the mouse monoclonal primary antibody. After further washes in TBS, the sections were incubated in 10% normal donkey serum (v/v), and 1% hydrogen peroxide (v/v) with 0.4% Triton X-100 (v/v) in TBS to block nonspecific binding of the secondary antibodies and to quench the endogenous peroxidases.

Brain sections were then incubated overnight in a cocktail containing primary antibodies against PRs, SRC-1, and SRC-2 in 1% normal donkey serum (v/v). A mouse monoclonal PR antibody directed against amino acids 922-933 of human PR (which correspond to amino acids 912-923 of mouse PR; 2.5 μg/ml; PR10A9, catalog #IM1408, Beckman Coulter), goat polyclonal SRC-1 targeted against the C terminus (amino acids 1355-1405) of mouse SRC-1 (1:200; M-20, catalog #SC6098, Santa Cruz Biotechnology) and rabbit polyclonal SRC-2 directed against the C terminus (amino acids 1400-1464) of human SRC-2 (1: 800; catalog #NB100-1756, Novus Biologicals) were used. The following day, sections were washed in TBS, then incubated for 90 min at room temperature in a cocktail of fluorescently labeled donkey anti-mouse (1:100; Alexa Fluor 594, Invitrogen), donkey anti-goat (1:100; Alexa Fluor 488), and donkey anti-rabbit secondary antibodies (1:100; Alexa Fluor 647) for the detection of PR, SRC-1, and SRC-2, respectively. The sections were washed in TBS, mounted onto Superfrost Plus slides (Fisher Scientific), coverslipped with Fluorogel (Electron Microscopy Sciences), and stored in the dark at 4°C until they were used for imaging.

The specificities of the SRC-1 and SRC-2 primary antibodies have been verified in mouse brain previously (Tognoni et al., 2011). The specificity of the mouse monoclonal PR primary antibody (PR10A9) was verified by both Western blot and immunohistochemistry. For the Western blot, recombinant mouse PRs from Sf9 cells were probed using PR 10A9, which revealed specific bands of the expected molecular mass for each isoform (data not shown). In further confirmation of its specificity in mouse brain, additional sections were colabeled with PR10A9 and a rabbit polyclonal PR antibody (1:500; catalog #A0098, DAKO) that has been used previously in the rodent brain (Quadros et al., 2008; López and Wagner, 2009), which showed similar labeling for both PR isoforms. Controls for this triple-label immunohistochemistry included the omission of the primary or secondary antibodies. In addition, a mouse monoclonal PR primary antibody (PR10A9) that was preadsorbed with a 20-fold molar excess of full-length mouse PR-B recombinant protein showed no labeling in tissue. Preadsorption controls for SRC-1 and SRC-2 have been performed previously (Tognoni et al., 2011).

### Imaging by confocal microscopy and analysis

The MPA, VMN, and ARC (Paxinos and Franklin, 2004, their Figs. 33, 46, and 46 of the mouse brain, respectively), which are rich in EB-induced PRs (MacLusky and McEwen, 1980; Davis et al., 1982; Blaustein and Turcotte, 1989; Pleim et al., 1989; Moffatt et al., 1998; Kudwa and Rissman, 2003; Tognoni et al., 2011), were analyzed by an experimenter who was blind to the treatment groups. Immunoreactive cells were imaged using a Leica laser-scanning confocal microscope (TCS SP5 II), equipped with argon, HeNe 594, and HeNe 633 lasers, and with Leica software (LAS version 2.7.3.9). All images were obtained under 400× magnification with the PLAN-APO oil-objective (numerical aperture, 1.25). AF 488, AF 594, and AF 647 fluorophores were excited using the Argon, HeNe 594, and HeNe 633 lasers, respectively. The gain and offset values for each laser were optimized for each chanel and kept constant within each brain region. For each brain region, images of 1-μm-thick optical sections were captured. Images were analyzed using the Nikon NIS Elements Advanced Research Software (version 3.22). A representative section from one side per animal was analyzed using uniforms region of interest for the MPA (total area, 171,722 μm^2^), VMN (total area, 86,560 μm^2^), and ARC (total area, 66,835 μm^2^), which were kept constant for each region across all animals.

Images from each of the three laser channels were merged to create a single RGB image and were calibrated using the scale bar on the image. A threshold for each RGB channel was set separately based on a scale of 0-255 to remove the background. The threshold settings were obtained by calculating the average background intensity of five random points from one representative animal per experimental group. The threshold was then set as a function of the SD of the background intensity and was kept constant within each region across animals. Any value below the threshold was considered to be background and was eliminated from the analysis. Size (6-100 μm) and circularity (0.65-1) restrictions were applied to clearly distinguish cells from the background. In each brain region, the number of immunoreactive cells and the average pixel intensity were collected for PR, SRC-1, and SRC-2 immunoreactivity.

### Statistical analysis

All statistical tests were conducted using the IBM SPSS Statistics Software (version 21). A two-way ANOVA (genotype and treatment) was run to examine differences in the number of PR-ir cells between groups. Tukey HSD *post hoc* tests were run to further compare differences between each group when a significant main effect or interaction was found. An unpaired two-tailed *t* test was used to examine the effect of treatment within each genotype.

One-way ANOVA was used to test the effect of genotype on mean PR-ir intensity in EB-treated groups. To examine the correlation between PR-ir intensity and the presence of SRC-1, SRC-2, or both coactivators, PR-ir cells were binned into the following four groups: (1) PR-only cells; (2) PR plus SRC-1 cells; (3) PR plus SRC-2 cells; and (4) PR plus SRC-1 and SRC-2 cells. The mean PR-ir intensities were calculated for each of the EB-treated genotypes, and a linear mixed-model regression analysis was used to examine the relationship between PR-ir intensity and the presence of none, one, or both coactivators among genotypes. When a significant main effect or interaction was detected, the mean PR-ir intensity of single-labeled PR cells (without SRC-1 or SRC-2 labeling) was compared with three other groups separately using an unpaired two-tailed *t* test for each region. For all statistical tests, differences were considered statistically significant at a probability of <0.05.

## Results

### Estradiol preferentially induces PR-A over PR-B in the hypothalamus of female mice

**Figure 1:**
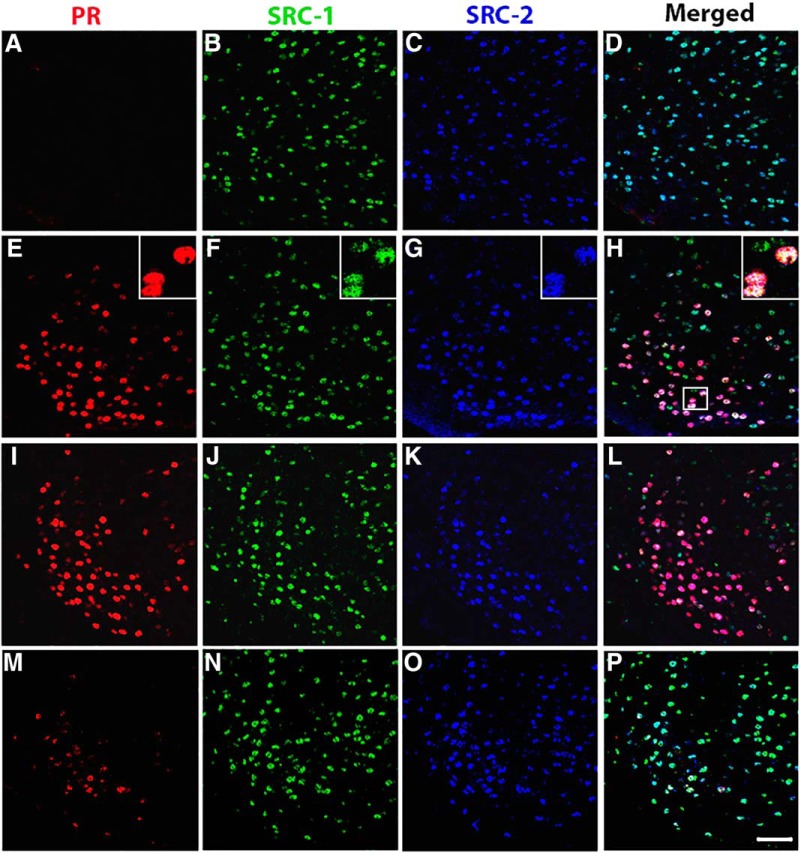
The majority of estradiol-induced PR-A or PR-B cells in the VMN of female mice coexpress SRC-1 and SRC-2. ***A–P***, Representative images taken from vehicle control wt mice (***A–D***) and estradiol-treated wt mice (***E–H***), PRBKO mice (that express PR-A only; ***I–L***), and PRAKO mice (that express PR-B only; ***M–P***). Insets show the magnified image of an area within the small square box. Magnification: images, 400×; insets, 630×. Scale bar, 50 μm.

**Figure 2:**
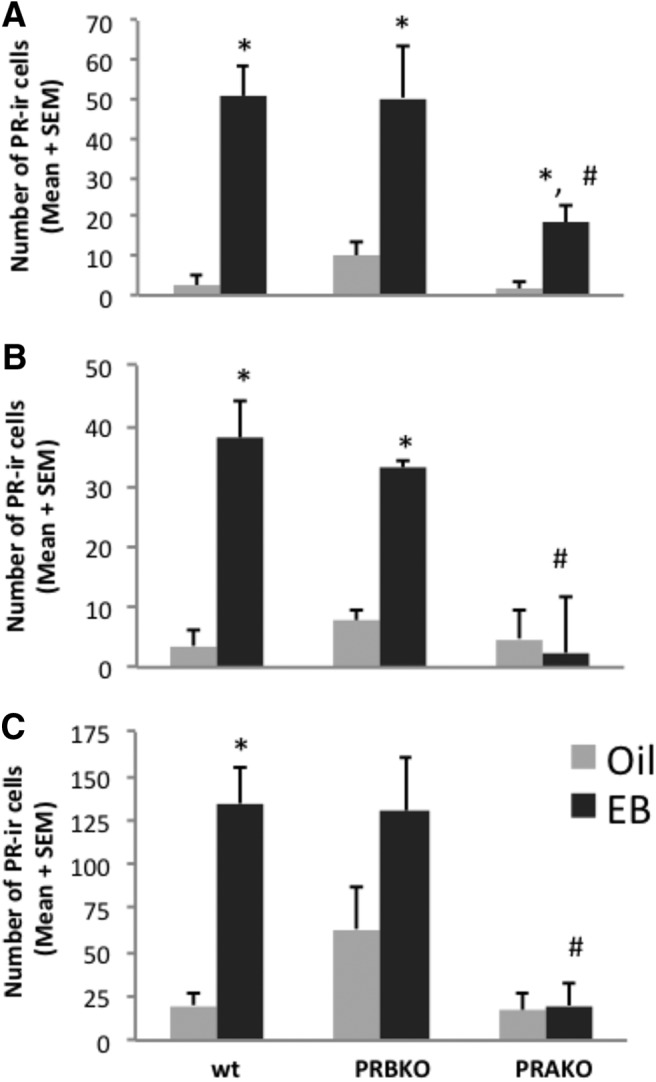
***A–C***, Estradiol induces PR-A and PR-B in a brain region-specific manner. Estradiol induces PR-A and PR-B in the VMN (***A***), but not in the ARC (***B***) or MPA (***C***), of PRBKO and PRAKO mice. *p < 0.05, for differences between vehicle and EB groups within each genotype; #*p* < 0.05, for differences between EB-treated genotypes.

To investigate the effects of EB on the expression of PR-A and PR-B in the hypothalamus, we compared the number of PR-ir cells of EB-primed PRAKO and PRBKO mice with that of vehicle controls. The wt mice were used to examine the effects of EB on total PR expression. Consistent with previous studies in mice and rats (MacLusky and McEwen, 1980; Parsons et al., 1980; Blaustein and Turcotte, 1989; Lauber et al., 1991; Turcotte and Blaustein, 1993; Moffatt et al., 1998; Kudwa and Rissman, 2003; Tognoni et al., 2011), EB induced PRs in the MPA, VMN, and ARC of wt mice (Fig. 1*E*), while little to no PR-ir cells were observed in vehicle controls (Fig. 1*A*). EB induced the expression of PRs in the hypothalamus of wt, PRAKO, and PRBKO female mice in a region- and isoform-specific manner. In the VMN, the number of PR-ir cells was increased in EB-primed mice in all three genotypes (*F*_(1,59)_ = 42.31, *p* = 0.001; Figs. 1*E*,*I*,*M*, 2*A*), but not in vehicle controls. Interestingly, EB-treated wt and PRBKO mice had a greater number of PR-ir cells compared with PRAKO mice (*F*_(2,59)_ = 5.03, *p* = 0.01; Fig. 2A), suggesting that PR-A is more strongly induced by EB than PR-B in the VMN.

In the ARC, EB increased the overall expression of PR (*F*_(1,55)_ = 18.02, *p* = 0.001). EB induced PRs in the wt and PRBKO mice (*F*_(2,55)_ = 7.19, *p* = 0.002), while no differences were observed between EB and vehicle groups in PRAKO mice. Within EB-treated groups, wt and PRBKO mice had more PR-ir cells compared with PRAKO mice (*F*_(2,55)_ = 6.92, *p* = 0.002; Fig. 2*B*). These data indicate that PR-A, but not PR-B, is EB-induced in the ARC.

In the MPA, EB increased PR expression (*F*_(1,60)_ = 10.97, *p* = 0.001), as observed in previous studies (MacLusky and McEwen, 1980; Blaustein and Turcotte, 1989; Moffatt et al., 1998; Kudwa and Rissman, 2003; Tognoni et al., 2011). Interestingly, EB-mediated PR induction was observed in wt mice (*F*_(2,60)_ = 3.25, *p* = 0.047), but not in PRAKO or PRBKO mice. However, a trend toward an increase in PR-ir cells was observed in EB-treated PRBKO mice compared with vehicle controls (Fig. 2*C*). Within EB-primed mice, wt and PRBKO mice contained more PR-ir cells compared with PRAKO mice (*F*_(2,60)_ = 8.41, *p* = 0.001), a finding that is similar to that observed in the VMN and ARC. These data indicate that EB induces PRs in the MPA of wt animals (when both PR isoforms are expressed) but does not increase either PR-A or PR-B alone in the isoform-specific knock-out mice.

**Figure 3:**
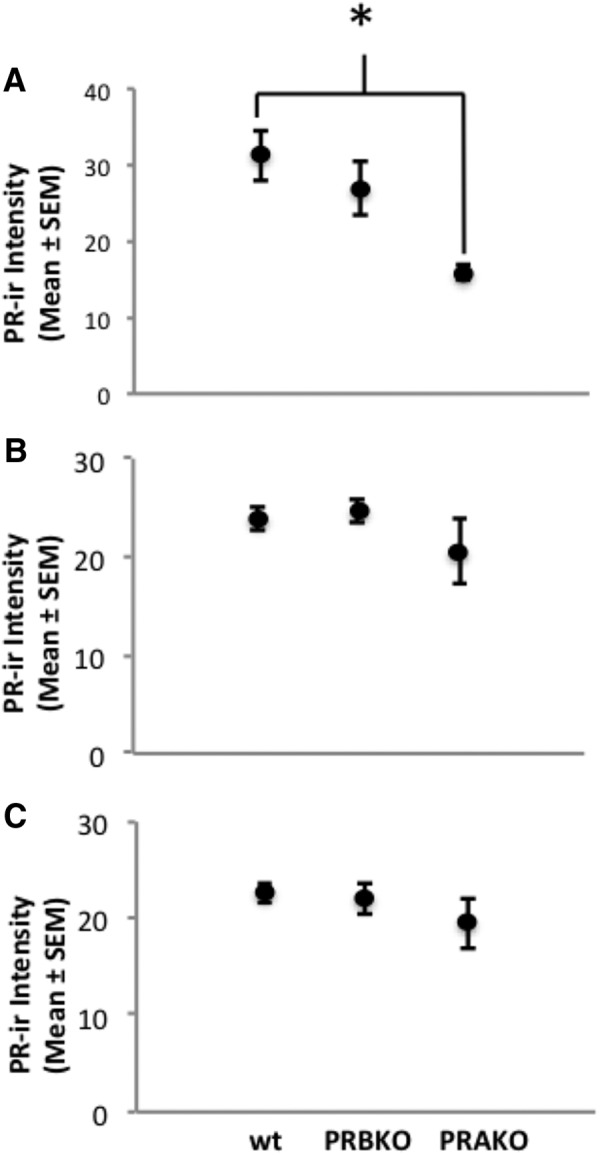
***A–C***, Immunostaining intensity of estradiol-induced PR is greater in wt mice than in PRAKO mice in the VMN (***A***), but not in the ARC (***B***) or MPA (***C***). *p < 0.05.

To investigate whether there was a difference between the relative amounts of PR-A and PR-B immunoreactive intensity, we examined the mean PR-ir intensity within cells in the VMN, ARC, and MPA of EB-treated wt, PRAKO, and PRBKO mice. In the VMN, mean PR-ir intensity was stronger in wt mice than in PRAKO mice (*F*_(2,29)_ = 6.265, *p* = 0.006; Fig. 3*A*). In the ARC and MPA, no differences were detected between genotypes (Fig. 3*B*,*C*).

### The majority of PR-A- and PR-B-containing cells in brain coexpress SRC-1 and SRC-2

We investigated the coexpression of the EB-induced PR isoforms and two members of the p160 family of coactivators (SRC-1 and SRC-2) in the brains of wt, PRAKO, and PRBKO mice. We found that SRC-1 and SRC-2 are widely expressed in the VMN, ARC, and MPA of female mice, as was observed in previous studies (Tognoni et al., 2011; Fig. 1). Moreover, the majority of EB-induced PR-ir cells coexpressed both SRC-1 and SRC-2 in wt mice in all the brain regions examined (Fig. 1*E–H*, Table 1), which is consistent with our previous findings (Tognoni et al., 2011). Only a smaller percentage of EB-induced PR-ir cells contained either SRC-1 or SRC-2 only (Table 1). In the VMN, fewer EB-induced PR-ir cells expressed SRC-2 than SRC-1 (Table 1), which is consistent with previous observations (Tognoni et al., 2011). A relatively small population of EB-induced PR-ir cells expressed neither of the coactivators. Together, these data extend findings from earlier studies (Apostolakis et al., 2002; Molenda et al., 2002; Molenda-Figueira et al., 2006) and provide neuroanatomical evidence that both SRC-1 and SRC-2 function in EB induction of PR-A and PR-B in the female mouse hypothalamus.

**Table 1:** The majority of estradiol-induced PR-ir cells (expressed as a percentage) coexpress both SRC-1 and SRC-2 in the VMN, ARC, and MPA of the hypothalamus in female mice

Brain region	Genotype	SRC-1 and SRC-2	SRC-1 only	SRC-2 only	Neither SRC-1 nor SRC-2
VMN	wt	62	22	4	12
	PRBKO	40	43	4	13
	PRAKO	64	31	0.3	5
ARC	wt	62	5	15	18
	PRBKO	52	17	15	16
MPA	wt	66	7	15	12

### EB-induced PR-A immunostaining is more intense in VMN cells expressing SRC-2

The effects of SRC-1, SRC-2, or both SRC-1 and SRC-2 on the mean PR-ir immunostaining intensity in EB-treated mice were examined. In the VMN, a positive correlation was observed between the presence of SRC-2 and PR-ir mean intensity (*F*_(1,70)_= 8, *p* = 0.001). More specifically, PRA-ir intensity (in PRBKO mice) was greater in cells expressing SRC-2 compared with PR cells expressing no coactivators (*p* = 0.04; Table 2), suggesting that this coactivator preferentially contributes to EB induction of PR-A. In support, in the VMN of wt mice, the PR-ir intensity in cells expressing SRC-2 (*p* = 0.03) or SRC-1 and SRC-2 (*p* = 0.02) was greater than that of PR cells that lacked coactivators (Table 2). Interestingly, no correlation between PR-ir intensity and the expression of SRC-1 or SRC-2 was observed in PRAKO mice.

**Table 2:** Estradiol-induced PR-ir cells in the VMN and MPA that express coactivators have greater immunostaining intensity than PR-ir cells that lack coactivators

Brain region	Genotype	PR only cells	PR cells SRC-1 and SRC-2	PR cells SRC-1	PR cells SRC-2
VMN	wt	22 ± 1.9	39 ± 6.7*	30 ± 3.6	37 ± 4.7*
	PRBKO	22 ± 3	36 ± 7.1	28 ± 4	34 ± 5.1*
	PRAKO	17 ± 3.1	16 ± 1.4	15 ± 0.9	16 ± 1.2
ARC	wt	24 ± 1.4	28 ± 2.1	24 ± 1.5	27 ± 1.2
	PRBKO	34 ± 8.9	25 ± 1.2	23 ± 1.2	29 ± 5.4
MPA	wt	18 ± 1.1	25 ± 1.8*	22 ± 1.3*	22 ± 1.3*

Values are given as the mean intensity ± SEM. In the VMN of wt and PRBKO mice, PR-ir cells that express SRC-2 have greater immunostaining intensity than PR-ir cells that lack SRC-2. In the MPA of wt mice, estradiol-induced PR-ir cells that express SRC-1 and/or SRC-2 have higher immunostaining intensity than PR-ir cells lacking these coactivators.

*p < 0.05, for differences between PR-only cell types.

In the MPA, the PR-ir intensity was positively correlated with SRC-1 (*F*_(2,40)_= 5.17, *p* = 0.01) and SRC-2 (*F*_(2,38)_ = 6.02, *p* = 0.005). In wt mice, EB-induced PR-ir intensity was greater in all cells expressing coactivators compared with PR cells lacking coactivators, suggesting a correlation between PR expression and the presence of SRC-1 or SRC-2 (Table 2). Because PR was not induced by EB in the MPA of PRBKO and PRAKO mice, the EB-induced PR-ir intensity of cell subtypes was not analyzed in these groups. In the ARC, there were no differences in EB-induced PR-ir intensity between the groups (Table 2). Together, these data suggest that the intensity of PR immunostaining correlates with the expression of SRC-1 and SRC-2 in an isoform- and brain region- specific manner. Neither EB treatment nor the presence of either PR isoform affected SRC-1 or SRC-2 expression in all regions examined (data not shown).

## Discussion

### PR-A is expressed more than PR-B in mouse brain

Using PR isoform-specific null mutant mice that express PR-A only (PRBKO) or PR-B only (PRAKO), we found that EB induces differential expression of the PR isoforms in the adult female hypothalamus. The expression of PR-A and PR-B was investigated in the VMN, ARC, and MPA, brain regions that contain estradiol-induced PR (MacLusky and McEwen, 1980; Davis et al., 1982; Blaustein and Turcotte, 1989; Pleim et al., 1989; Moffatt et al., 1998; Kudwa and Rissman, 2003; Tognoni et al., 2011) and are important for female reproduction (Conneely et al., 2001, Mulac-Jericevic and Conneely, 2004; Brinton et al., 2008; Mani and Blaustein, 2012). Consistent with previous studies (Moffatt et al., 1998; Kudwa and Rissman, 2003; Kudwa et al., 2004; Tognoni et al., 2011), EB dramatically induced PR in these three brain regions in wt mice. Interestingly, in PRAKO and PRBKO mice treated with EB, we found that the PR-A isoform is dominantly expressed over PR-B in the VMN, ARC, and MPA. A similar number of PR-ir cells were present in PRBKO mice (express PR-A only) and wt mice, suggesting that PR-A constitutes the majority of total EB-induced PR in the hypothalamus of wt females. These results are supported by previous reports that PR-A in brain contributes more to the expression of female reproductive behavior than PR-B in mice (Mani et al., 2006). In contrast to mice, in female rats, while both PR isoforms are EB-induced in brain (Guerra-Araiza et al., 2002, 2003; Scott et al., 2002), sexual receptivity appears to be primarily mediated through PR-B (Guerra-Araiza et al., 2009). Together, these findings reveal species-specific differences in the expression and function of the PR isoforms.

#### Brain region-specific expression of PR isoforms

In the present study, a difference was detected in the expression of PR isoforms among the VMN, ARC, and MPA. In the VMN only, both PR-A and PR-B were induced by EB. Interestingly, EB induced only PR-A in the ARC and neither isoform in the MPA. The present data provide evidence that the two PR isoforms are differentially induced and provide a mechanism by which progestin action can be fine-tuned in these brain regions that are involved in female reproductive physiology and behavior.

Similar to the present findings in the hypothalamus, PR-A is the more prevalent isoform in mouse uterus, where it is required for normal ovarian and uterine development and function (Conneely et al., 2003). Similarly, PR-A is more abundantly expressed than PR-B in the human and rat uterus (Ilenchuk and Walters, 1987; Mangal et al., 1997; Mote et al., 2000). In the mammary gland, although PR-B mediates normal alveolar development during pregnancy (Conneely et al., 2001), PR-A is necessary for ductal side branching and is strongly regulated by estrogens in adult females (Aupperlee and Haslam, 2007), which is consistent with the present results in the hypothalamus. Interestingly, in some human cell lines PR-B is a stronger transcriptional activator than PR-A, and PR-A represses PR-B-mediated transcription in a hormone-dependent manner (Vegeto et al., 1993). Together with the present data, PR-A has a predominant role in mouse brain and in human, rat, and mouse uterus, while both isoforms function in mammary gland development, suggesting tissue-specific roles for the PR isoforms.

#### Potential regulatory mechanisms for differential PR isoform expression in brain

While the specific mechanisms regulating differential expression of PR isoforms in the female mouse hypothalamus are not completely understood, differences in the *Pgr-A* and *Pgr-B* gene promoters may play a role. The *Pgr* gene transcription is mediated by distinct estrogen-inducible promoters to produce the PR isoforms (Kastner et al., 1990). The *Pgr-A* promoter, but not the *Pgr-B* promoter, contains an imperfect but functional estrogen response element in mice and rats (Kastner et al., 1990; Hagihara et al., 1994; Xu et al., 2004). In addition, transcription factors such as Sp1 can facilitate ER-mediated PR induction by interacting with *Pgr* promoters (Sriraman et al., 2003; Xu et al., 2004; O'Brien et al., 2006). In the future, it will be important to use the PR isoform-specific knock-out mice to explore the role of Sp1 and other transcription factors in the estradiol induction of the two PR isoforms in brain. Furthermore, with recent advances in genome-wide analysis of ER binding regions, additional ER binding sites that influence PR expression have been identified in distal regulatory regions of the gene (Carroll et al., 2006; Sharma et al., 2014). It will be important in future studies to investigate possible functions of the promoters and distal regulatory elements of the *Pgr* gene using the PR isoform-specific knock-out mice.

### Coexpression of PR-A and PR-B with SRC-1 and SRC-2 in brain

#### Role of SRCs in EB-mediated PR expression

SRC-1 and SRC-2 modulate nuclear receptor action to regulate energy homeostasis, circadian rhythm, sexual differentiation of the brain, stress, and reproductive behavior (Auger et al., 2000; Charlier et al., 2006; Molenda-Figueira et al., 2006; Winnay et al., 2006; Lachize et al., 2009; Tetel and Acharya, 2013; Zhu et al., 2013; Stashi et al., 2014a,b). For example, we as well as others have previously demonstrated that SRC-1 and SRC-2 function in EB-mediated induction of PR in the hypothalamus and in ER- and PR-dependent aspects of reproductive behavior (Apostolakis et al., 2002; Molenda et al., 2002; Molenda-Figueira et al., 2006). To understand the potential role of coactivators in EB induction of PR-A and PR-B, we investigated the coexpression of each PR isoform with SRC-1 and SRC-2. Consistent with previous findings (Tognoni et al., 2011), the majority of EB-induced PR-ir cells coexpress both SRC-1 and SRC-2 in the VMN, ARC, and MPA of wt female mice. Furthermore, the present results reveal that most EB-induced PRA-ir (in PRBKO mice) and PRB-ir (in PRAKO mice) cells coexpress both SRC-1 and SRC-2 in these three brain regions, suggesting that both of these coactivators contribute to the ER-mediated induction of both PR isoforms. Since virtually all EB-induced PR cells in these brain regions express ERα (Blaustein and Turcotte, 1989; Warembourg et al., 1989), these PR-A and PR-B cells contain ERα and both coactivators (SRC-1 and SRC-2). While a smaller population of PR-A or PR-B cells expressed either SRC-1 or SRC-2 only, few others expressed neither SRC-1 nor SRC-2. It is possible that the triple-label immunofluorescent technique did not detect low levels of the coactivators in these PR-A or PR-B cell populations. Alternatively, ER-mediated induction of a distinct population of PRs in cells lacking SRC-1 and SRC-2 could be regulated by other coactivators or by coactivator-independent pathways (Tetel, 2009; Tetel and Acharya, 2013; Borgquist et al., 2014). Finally, not all coactivator-expressing cells contained PRs, suggesting that these coactivators function with other steroid receptors, such as receptors for androgens or glucocorticoids in brain (Tetel and Acharya, 2013). Together, the present data provide neuroanatomical evidence that SRC-1 and SRC-2 potentially contribute to the ER-mediated induction of the PR isoforms in brain.

To test the hypothesis that SRC-1 and SRC-2 have differential roles in the EB induction of the hypothalamic PR isoforms, the intensity of PR-A and PR-B immunostaining was measured in PR-isoform-specific null mutant mice. PR-ir intensity was correlated with the expression of coactivators in a brain region-dependent manner. In the VMN of wt and PRBKO mice (expressing PR-A only), SRC-2 correlated with stronger PR-ir intensity, suggesting that SRC-2 contributes preferentially to EB induction of PR-A. In the MPA, both SRC-1 and SRC-2 were associated with greater PR-ir intensity in EB-treated wt mice. These data suggest that SRC-1 and SRC-2 contribute differentially in the ER-mediated induction of PR-A and PR-B in a brain region-specific manner. In future studies, it will be important to directly test this concept by altering coactivator expression in PR isoform-specific null mutant mice.

Finally, while the present findings do not directly test function, they do provide neuroanatomical evidence suggesting that SRC-1 and SRC-2 may act with the PR isoforms in brain. In support, antisense to SRC-1 in the VMN decreases PR-dependent female reproductive behavior (Molenda-Figueira et al., 2006). Moreover, our previous findings show that PR-A and PR-B physically interact with SRC-1 and SRC-2 from hypothalamus in a ligand- and isoform-dependent manner (Molenda-Figueira et al., 2008; Yore et al., 2010). It will be important in future studies to investigate the function of these coactivators on the action of each PR isoform in brain and behavior using the PRAKO and PRBKO mice.

#### Summary

A variety of studies indicate that PR-A and PR-B in brain and other tissues have distinct functions in humans and rodents (Vegeto et al., 1993; Wen et al., 1994; Conneely et al., 2001, 2003; Mani et al., 2006; Guerra-Araiza et al., 2009). The present and novel results that PR-A is expressed more than PR-B in reproductively relevant brain regions provide a mechanism by which progestin action can be modulated in brain. Understanding how PR-A and PR-B function in these brain regions is critical to understanding PR action in brain and behavior. Furthermore, these results suggest that SRC-1 and SRC-2 have distinct roles in the ER-mediated induction of the PR isoforms among brain regions, providing a potential mechanism for achieving this differential expression of PR-A and PR-B. A better understanding of the mechanisms through which nuclear receptor coactivators, including SRC-1 and SRC-2, regulate PR expression and function in hypothalamus in a region- and isoform-dependent manner will enhance our knowledge of steroid-dependent neural processes such as neurogenesis, neuroprotection, sexual differentiation, learning and memory, energy homeostasis, and reproductive behavior and physiology (Nilsen and Brinton, 2002; Brinton et al., 2008; McCarthy and Nugent, 2013; Tetel and Acharya, 2013; Bless et al., 2014; Arevalo et al., 2015; Duarte-Guterman et al., 2015; Frick, 2015; López and Tena-Sempere; 2015; Willing and Wagner, 2015).

## Synthesis

The decision was a result of the Reviewing Editor Jeffrey Blaustein and the peer reviewers coming together and discussing their recommendations until a consensus was reached. A fact-based synthesis statement explaining their decision and outlining what is needed to prepare a revision is listed below.

The reviewers and I agree that, although it is a purely descriptive study, it is worthy of publication. The one thing that is missing is a persuasive argument to eNEURO readers, as opposed to ENDO readers, why they should be interested. What does it add to our understanding of Neuroscience?

Reviewer 1 comments:

The authors have made very minor changes to the manuscript that do not address my primary concerns about the novelty and significance of the findings. It's fine to add "for the first time" to the text, but the text has not been altered to directly address why readers of eNeuro should care about the findings. What is the broad significance of the study to neuroscience? Why are these descriptive findings important? Without this context, it is difficult to see why these findings would be of interest to the broad readership of the journal rather than that of a specialty journal.
